# What the Health? Information Sources and Maternal Lifestyle Behaviors

**DOI:** 10.2196/10355

**Published:** 2019-07-05

**Authors:** Emilie Mass Dalhaug, Lene Annette Hagen Haakstad

**Affiliations:** 1 The Norwegian School of Sport Sciences Department of Sports Medicine Oslo Norway

**Keywords:** pregnancy, physical activity, gestational weight gain, diet, prenatal care, behavior

## Abstract

**Background:**

Regular physical activity (PA), adequate gestational weight gain (GWG), and healthy eating are important for the long-term health of both mother and baby. Hence, it is important that women receive current and updated advice on these topics and are encouraged to adopt a healthy lifestyle during pregnancy.

**Objective:**

The aim of this study was to investigate the main information sources among pregnant women regarding PA, GWG, and nutrition as well as to evaluate how these information sources may affect their health behaviors.

**Methods:**

A cross-sectional study design, comprising an electronic questionnaire, was distributed to 2 antenatal clinics, as well as pregnancy-related online chat forums and social media. The inclusion criteria were ≥18 years, ≥20 weeks gestation, and able to read and write Norwegian. In total, 150 pregnant women answered the questionnaire, which was a mix of 11-point Likert scales, close-ended questions, and semi–close-ended questions with the option to elaborate. The relationship between information sources and selected variables, including health behaviors and descriptive variables, were assessed by logistic regression, linear regression, or chi-square as appropriate (*P*<.05).

**Results:**

Mean age (years), gestation week, and prepregnancy body mass index (kg/m^2^) were 31.1 (SD 4.3), 30.6 (SD 5.9), and 24.2 (SD 4.2), respectively. More than eight out of 10 had received or retrieved information about nutrition (88.7%, 133/150) and PA (80.7%, 121/150), whereas 54.0% (81/150) reported information on GWG. When combining all 3 lifestyle factors, 38.5% had retrieved information from blogs and online forums and 26.6%, from their midwife or family physician. Women who reported the internet and media as their primary source of information on weight gain had increased odds of gaining weight below the Institute of Medicine (IOM) guidelines compared with gaining within the guidelines (odds ratio [OR] 15.5, 95% CI 1.4-167.4; *P*=.02). Higher compliance with nutritional guidelines was seen among those who cited the internet and media as their main source of information on nutrition (beta=.7, 95% CI 0.07-1.3; *P*=.03). On the other side, receiving advice from friends and family on weight gain was significantly associated with gaining weight above the IOM guidelines compared with gaining within the guidelines (OR 12.0, 95% CI 1.3-111.7; *P*=.03). No other associations were found between information sources and health behaviors.

**Conclusions:**

The small number of health professionals giving information and the extensive use of internet- and media-based sources emphasize the need to address the quality of internet advice and guide women toward trustworthy sources of information during pregnancy. The association between information sources and PA, GWG, and nutrition requires further research.

## Introduction

Regular physical activity (PA), adequate gestational weight gain (GWG), and healthy eating may lower the incidence and severity of serious conditions associated with pregnancy, including gestational diabetes mellitus [1-3], pregnancy-induced hypertension [2], preterm birth [4], macrosomia [1,4], and small for gestational age infants [4,5]. In addition, daily exercise for the pelvic floor muscles may prevent and treat urinary incontinence [6]. Hence, it is important that women receive current and updated advice on these topics and are encouraged to adopt a healthy lifestyle during pregnancy [7].

Research show that pregnant women retrieve health information from a variety of sources, including the internet, books, family and friends, parenting magazines, blogs, online forums and health professionals [8-10]. Of these, the internet and media and health professionals are often cited as the most helpful and informative sources of information [8,10]. On the contrary, a meta-analysis showed that health websites often lacked accuracy and that it was difficult to find high quality sites [11]. Others have found that pregnant women may perceive advice from family physicians and midwives confusing, vague, contradictory, and frequently changing [9,12-14]. In addition, research shows that most health care providers, regardless of medical training, lack knowledge and awareness of the American College of Obstetricians and Gynecologists [7] PA guidelines [13,15] and the Institute of Medicine (IOM) [16] GWG guidelines [15,17,18]. Furthermore, the majority of pregnant women being counselled about weight gain report that the advice is generally discordant with the guidelines [8,13,19,20].

In previous studies investigating pregnant women’s information sources on PA, GWG, and/or nutrition [8-10,13,14,19,21-27], the population sizes were generally small (N=17-60) [9,13,14,21,23,26] and information sources were rarely the main outcome [9,13,19,22-26]. In addition, studies of moderate methodological quality and sufficient population sizes (N=350-368) have recruited women in the postpartum period, limiting the results to the women’s memory [10,27], or halfway through pregnancy (mean 20.8 weeks) [8]. Hence, there is limited evidence investigating pregnant women’s information sources regarding PA, GWG, and nutrition at late gestation. To our knowledge, this study is also the first to evaluate how different information sources may influence PA, GWG, and nutritional habits among pregnant women. Thus, the aim of this study was to investigate the main information sources among pregnant women in Scandinavia with regard to PA, GWG, and nutrition and evaluate how these information sources may affect their health behaviors.

## Methods

### Study Design

The present project was a cross-sectional study conducted in Oslo, Norway, from February to August 2016. Pregnant women were asked to fill an electronic questionnaire investigating their health behaviors, as well as information sources with regard to PA, GWG, and nutrition. The study was reviewed by the Regional Committee for Medical and Health Research Ethics (REK 2015/1941 A), who concluded that, according to the act on medical and health research (the Health Research Act 2008), the study did not require full review by REK. The study was approved by the Norwegian Social Science Data Service (NSD 45111).

### Participants

Enrolment was limited to women living in Oslo, ≥18 years, ≥20 weeks gestation, and being able to read and write Norwegian.

### Procedures

To ensure a representative sample with regard to different ethnicities, age groups, and socioeconomic backgrounds, all antenatal clinics in Oslo (n=18), both urban and rural, were invited to participate. However, due to ongoing research projects only 2 agreed to distribute questionnaires to their pregnant patients. Hence, we needed to recruit participants from other arenas. We chose to spread the link to the online survey through advertisements on Facebook and Instagram, as well as through various pregnancy-related online chat forums and the university website. The advertisement on Facebook and Instagram was not limited to pregnant women but targeted women living in Oslo. The internet-based questionnaire was active between June 1 and August 15, 2016.

### Outcome Measures

The survey items used to answer our research questions were derived from a multidimensional survey that assessed pregnant women’s information sources, PA level, nutritional habits, and GWG. The survey also investigated social support, motives, and barriers for being physically active, as well as pregnancy complaints and quality of life. The multidimensional electronic survey contained 101 questions and was developed using existing and validated questions [28-30], as well as some newly developed questions suitable to the purpose of this study. The current analysis focused on pregnant women’s information sources and their lifestyle behaviors. Questions were a mix of 11-point Likert scales, close-ended questions, and semi–close-ended questions with the option to elaborate. The questionnaire was piloted for comprehensibility of questions and answer options among 23 pregnant women and was revised accordingly. Below are the questions used to answer this study’s research questions (Table 1). A full questionnaire list in Norwegian may be provided upon request.

**Table 1:** Dimensions assessed and main variables and questions used to answer the research questions.

Dimensions assessed	Main variables and questions used	Reference
Sociodemographic characteristics	Age, gestation week, parity, marital status, place of residence, country of birth, educational level, occupation, and number of antenatal consultations.	Developed for this project
Anthropometry and knowledge of GWG^a^ guidelines	Participants were asked to state their height, pre-pregnancy weight, and current GWG.^b^ Also, women were asked whether they were familiar with the IOM^c^ GWG table.	Developed for this project
Physical activity	Assessed using the question: The health authorities recommend all pregnant women to perform moderate-intensity aerobic physical activity (activities that take moderate physical effort and make you breathe somewhat harder than normal, such as brisk walking, housework etc) for a minimum of 30 minutes five days a week. With this in mind, would you characterize yourself as physically active a) prepregnancy and b) in your current gestation week? Response options: “Yes”, “No” or “I don’t know”.	Based on the American College of Obstetricians and Gynecologists recommendations [7]
Compliance with nutritional guidelines	Assessed using the question: “The Norwegian directorate of health recommend a balanced and varied diet, comprised of whole grain products, vegetables, fruits and berries, lean dairy products, fish, legumes and nuts, while also limiting the amount of processed meats, red meat and foods high in saturated fat, sugar and salt [31]. With this in mind, how would you characterize your diet in your current gestation week?” The participants rated their diet on a scale from 0-10, where 0 represented “Very poor” and 10 represented “Very good”.	Sagedal et al [28]
Information sources on PA^d^/GWG/nutrition	Assessed using the questions: “From where did you receive/retrieve information on PA/GWG/nutrition?” and: “Which of the information sources had the greatest impact on your PA/GWG/nutrition?” Response options (participants were able to choose more than one information source): “Midwife”, “Family physician”, “Blogs and Internet forums”, “Parenting magazines”, “Books and information pamphlets”, “Family and friends”, “I have not received/retrieved information/advice” and “Other”.	Developed for this project
Advice on PA	Assessed using the question: “Have you received any of the following advice on PA?” Response options: “Maintain the same level of PA as prepregnancy”, “Increase PA/exercise”, “Reduce PA/exercise”, “Avoid PA/exercise” and “Other”.	Developed for this project
Advice on GWG	Assessed using the question: “How much (in kg) have the information sources indicated that your total GWG should be?”	Developed for this project

^a^GWG: gestational weight gain.

^b^Prepregnancy height and weight were used to calculate prepregnancy body mass index (BMI). BMI categories and gestational weight gain ranges were consistent with the World Health Organization’s guidelines [32] and the guidelines from the IOM [16].

^c^IOM: Institute of Medicine.

^d^PA: physical activity.

### Statistical Analyses

All statistical analyses were performed using IBM SPSS Statistical Software version 21.0 for Windows. Background variables and information sources are presented as frequencies, percentages, or means with standard deviation.

For the purpose of analysis, we divided the participants into 3 groups based on the information source perceived to mostly impact maternal health behavior:

1. Internet and media (including blogs and online forums, parenting magazines and books, and information pamphlets).
2. Health professionals (including midwives and family physicians).
3. Friends and family.

Whether a woman had gained weight below, within, or above the GWG guidelines was calculated using mean recommended weight gain in the first trimester (1.5 kg), adding the mean recommended number of grams per week multiplied by the number of weeks the woman was pregnant above the first trimester [16]. Good nutritional habits and compliance with nutritional guidelines was defined as a score ≥7 on an 11-point scale. The relationship between information sources and selected variables, including health behaviors and sociodemographic variables, was assessed by logistic regression, linear regression, or chi-square as appropriate. The level of statistical significance was set at *P*<.05.

## Results

Participant characteristics are shown in Table 2. Responses were received from 275 pregnant women, 244 of whom were recruited through social media and 31, recruited through antenatal clinics. The analysis included only the results from the 150 participants who fully completed the questionnaire and provided data on information sources and health behaviors. The participants were residents in both urban and rural parts of Oslo. The age ranged from 19 to 45 years with a mean of 31.1 (SD 4.3) years. The mean gestation week was 30.6 (SD 5.9) and mean prepregnancy body mass index (BMI) was 24.2 (SD 4.2) kg/m^2^. The mean number of antenatal consultations was 5.2 (SD 2.7; range 1-15).

Almost 90% of women reported that they were physically active for a minimum of 150 min of moderate intensity each week before pregnancy. This number decreased to less than 50% in the current gestation week (Table 2).

As shown in Table 3, nearly 65% of the women had gained weight outside the GWG guidelines. About half the participants (48.9%) had knowledge of the IOM table for recommended weight gain (Table 3). Binary logistic regression analyses revealed that weight gain was not significantly associated with knowledge of GWG guidelines (Table 4). The odds of gaining weight within recommended levels were not significantly different among participants knowing and not knowing about the guidelines (Table 4). Furthermore, the odds of knowing the GWG guidelines did not differ among those gaining weight below or above the recommended level as compared with those gaining within the recommended levels (Table 4). The low number of participants within the different categories limited adjustments of the statistical models. Nevertheless, the results in Table 4 did not change to any appreciable extent when we adjusted for parity, education level, and prepregnancy BMI (data not shown).

Perceived good nutritional habits and compliance with the nutritional guidelines was reported by 65.3% (98/150) of the women.

**Table 2:** Participant characteristics (N=150).

Characteristics		Value, n (%)	
**Parity**			
	Primiparous		91 (60.7)
	Multiparous		59 (39.3)
**Marital status**			
	Married or living together		147 (98.0)
	Other		3 (2.0)
**Country of birth**			
	Norway		130 (86.7)
	Other		20 (13.3)
**Education**			
	<4 years college or university		54 (36.0)
	≥4 years college or university		96 (64.0)
**Employment status**			
	Employed or student		144 (96.0)
	Not employed		6 (4.0)
**Physically active**			
	Prepregnancy		132 (88.0)
	During pregnancy		73 (48.7)
**Prepregnancy body mass index category**			
	Underweight		2 (1.3)
	Normal weight		102 (68.4)
	Overweight		28 (18.7)
	Obese		17 (11.4)
**Prenatal care provider**			
	Family physician		25 (16.7)
	Midwife		43 (28.7)
	Shared care^a^		75 (50.0)
	Other		7 (4.7)

^a^Antenatal care shared between midwife and family physician.

**Table 3:** Women gaining within, below, or above the Institute of Medicine (IOM) recommendations (n=139). Data are presented in frequency (n), percentage (%), mean (SD) kg below and above recommendations and knowledge of IOM guidelines.

Variable	n (%)	mean (SD)	Knowledge of IOM^a^ guidelines, n (%)
Within recommendations	51 (36.7)	—^b^	27 (19.4)
Below recommendations	37 (26.7)	−2.6 (2.2)	14 (10.0)
Above recommendations	51 (36.7)	+3.0 (2.4)	27 (19.4)

^a^IOM: Institute of Medicine.

^b^Not applicable.

**Table 4:** Statistics associated with (A) the odds of gaining weight within recommended levels for participants having knowledge of the Institute of Medicine (IOM) guidelines compared with participants without knowledge of the IOM guidelines and (B) the odds of having knowledge of the IOM guidelines for participants gaining weight below and above the recommended levels compared with participants gaining within the recommended levels.

Variable		n	Odds ratio (95% CI)	*P* value
**A**				
	Not knowing the guideline	68	Ref^a^	—^b^
	Knowing the guideline	68	1.29 (0.64-2.59)	.48
**B**				
	Within recommendations	51	Ref	—
	Below recommendations	37	0.54 (0.23-1.29)	.17
	Above recommendations	51	1 (0.45-2.20)	1.00

^a^Ref: statistical reference group.

^b^Not applicable.

### Information Sources

Most women reported multiple information sources on PA, GWG, and nutrition (Table 5). More than 8 out of 10 had received or retrieved information about nutrition (88.7%, 133/150) and PA (80.7%, 121/150), whereas 54.0% (81/150) reported information on GWG. When combining all 3 lifestyle factors, 38.5% had retrieved information from blogs and online forums and 26.7% from their midwife or family physician (Table 5). Books and information pamphlets were the most frequent information source on nutrition, reported by 48.0% (72/150).

Pearson’s chi-square tests revealed that across all 3 lifestyle factors, significantly more women reported internet and media sources than health professionals as the information source with the most impact on their health behavior (PA: chi-square=23.25; *P*<.001; GWG: chi-square=38.13; *P*<.001; nutrition: chi-square=67.11; *P*<.001; Table 6). Significantly more women also reported family and friends to be the most important source of PA information compared with health professionals (chi-square=7.31; *P*=.007), but there was no difference between family and friends and health professionals with regard to GWG (chi-square=2.14; *P*=.14) and nutrition (chi-square=2.75; *P*=.097; Table 6).

**Table 5:** Pregnant women’s information sources on physical activity, gestational weight gain, and nutrition.

Information sources	Physical activity, %	Gestational weight gain, %	Nutrition, %
Blogs and online forums	42.7	32.0	40.7
Books and information pamphlets	32.0	22.0	48.0
Parenting magazines	20.7	10.0	20.7
Friends and family	27.3	7.3	22.7
Midwife	30.7	18.0	35.3
Family physician	28.7	14.0	34.0
Other	10.0	2.7	7.3
I have not received or retrieved information	19.3	46.0	11.3

**Table 6:** Information sources with the most impact on health behavior. Participants were divided into 3 groups based on the information source perceived to mostly impact maternal health behavior.

Information sources	Physical activity, %	Gestational weight gain, %	Nutrition, %
Internet and media	54.1	63.2	68.0
Friends and family	27.1	8.8	8.0
Health professionals	18.8	28.1	24.0

When examining sources of information across sociodemographic groups, including age, education level, employment status, parity, number of children, marital status, and country of birth, the only significant association was between high education and friends and family as the main information source on nutrition. The odds of reporting friends and family as the most important source of information with regard to nutrition during pregnancy was 67% lower among women with ≥4 years of college or university education compared with women with <4 years of college or university education (odds ratio [OR] 0.33, 95% CI 0.13-0.85; *P*=.02).

### Advice Consistent With Guidelines

Irrespective of the information source, 32.8% and 27.3% of participants had received advice inconsistent with the GWG guidelines [16] and PA guidelines [7], respectively. Less than one-third of women (29.3%, 44/150) had received specific advice on PA from a midwife or family physician. Of these, 25.0% (11/150) had received advice inconsistent with the present PA guidelines. With regard to GWG, 13.3% (20/150) reported receiving specific advice from a health professional, and half of these had received advice inconsistent with the GWG guidelines.

### Impact of Information Sources on Women’s Health Behaviors

No significant associations were observed between the 3 groups of information sources and the odds of meeting the PA guidelines.

Binary logistic regressions on the impact of information sources on GWG revealed that choosing internet and media or health professionals as the most important source on GWG information increased the odds of gaining below the guidelines compared with gaining within the guidelines (internet and media: n=40, OR 15.5, 95% CI 1.4-167.4; *P*=.02; health professionals: n=40, OR 7.9, 95% CI 0.7-83.8; *P*=.08). Choosing friends and family as the most important source on GWG information increased the odds of gaining above the guidelines compared with gaining within (n=56, OR 12.0, 95% CI 1.3-111.7; *P*=.03). Otherwise, no significant relationships or interaction effects were observed.

Linear regressions revealed that choosing the internet and media as the most important information source on nutrition was associated with an increase of 0.7 on the 11-item scale representing compliance with nutritional recommendations (n=133; 95% CI 0.07-1.3; *P*=.03). This association remained significant when controlling for self-reported diet before pregnancy (*P*=.03). No significant associations were found between the categories, family and friends or health professionals and compliance with nutritional guidelines.

## Discussion

### Principal Findings

To our knowledge, this was the first study investigating the relationship between pregnant women’s information sources and their health behaviors. Consistent with previous research, the most common sources of information were the internet and media [8,33]. About one-fourth of women reported receiving information on PA, GWG, and nutrition from their midwife or family physician. Reporting the internet and media as the most important source increased the odds of gaining weight below the GWG guidelines but was also associated with higher compliance with nutritional guidelines. The category, friends and family, was significantly associated with gaining above the GWG guidelines.

Our recruitment method may be an important reason for the large proportion of women choosing the internet and media as preferred information sources and may have skewed the results. On the contrary, our results correspond with other studies, recruiting women in the first hospital antenatal visit [8,27]. A qualitative study has found that women turn to the internet to gather information before meeting with a health professional and afterward to obtain more information [23]. Social media was also considered an arena for socializing and sharing experiences with other pregnant women and mothers [23]. In this study, we have not thoroughly investigated the quality of the internet advice, but a meta-analysis of health website evaluations concluded that the advice often lacked accuracy and that it was difficult to find high quality sites [11]. Hence, it is important that women are guided toward trustworthy online resources during pregnancy.

According to the Norwegian guidelines for antenatal care, all prenatal patients should receive lifestyle counselling, including advice on PA, GWG, and nutrition on the first prenatal visit [34]. Consistent with other studies, only one-fourth of women in this study reported receiving advice from their midwife or family physician on these topics [8,9,13,14,19,20]. Moreover, only 10 participants received advice consistent with the GWG guidelines. Although others have also reported low numbers (5.2%-12.0%) with regard to information on GWG from health professionals [8,20], this is in contrast to research showing that receiving advice from a health professional increases the likelihood of gaining weight within the guidelines [27,35]. Hence, interventions are needed to increase the percentage of health professionals who accurately advise women on PA, GWG, and nutrition during pregnancy.

### Impact on Women’s Health Behaviors

Stating the internet and media as the most important sources on GWG information significantly increased the odds of gaining below the GWG guidelines. Hicks and Brown [36] found that time spent on social media was associated with body dissatisfaction among pregnant women. In this study, no association between negative body image and choosing the internet and media as the most important sources of information was found (data not shown). Yet, the way internet and media idealize a slender pregnant woman with a neat bump [36] may have influenced the women’s GWG.

Reporting friends and family as the main information source on GWG was associated with gaining above the GWG guidelines. Public health guidelines on GWG have been revised several times over the past decades [16,37] and friends and family might not have knowledge of the current guidelines, and therefore give incorrect advice. However, only 10 women in this study considered friends and family to have the most impact on GWG.

The most frequently reported information source on nutrition was books and information pamphlets, highlighting the importance of this media for dietary information. The Norwegian Directorate of Health [31] distributes an information pamphlet with updated nutritional guidelines, available at antenatal clinics as well as on the internet. In this study, we observed that compliance with nutritional guidelines was significantly associated with reporting the internet and media as the primary information source. This association remained significant after controlling for self-reported diet before pregnancy.

### Strengths and Limitations

This is one of the largest studies investigating pregnant women’s information sources and the first to explore how different information sources may impact 3 distinct but importantly related health topics: PA, GWG, and nutrition. In addition, the questionnaire used covered a broad range of factors that could possibly explain women’s health behaviors and was based on previously validated questions and questions used in similar studies [28-30,38]. Furthermore, the use of an electronic questionnaire is time efficient and cost-effective [39]. On the contrary, the internet recruitment made it difficult to determine the response rate. Moreover, the results of this study cannot be generalized owing to the recruitment of participants through social media and an overrepresentation of highly educated Nordic Caucasian women. In addition, a much higher percentage of participants reported meeting the PA guidelines compared with previous reports [30,40]. Pregnant women answering such a questionnaire may be more interested and more attentive to PA than nonparticipants, thus introducing the problem of selection bias. Our initial approach was to recruit participants through all 18 antenatal clinics in Oslo. However, only 2 decided to participate. Instead of recruiting women through social media and thus compromising the generalizability of the results, we should have explored the possibility of recruiting women through endocrine clinics or specialized clinics. Yet, the findings regarding women’s information sources are consistent with other research [8,27]. Moreover, the use of a nonvalidated question to assess PA and dietary quality and the lack of questions evaluating predictors of health behavior change makes the results less reliable. All information was self-reported and therefore subjective to social desirability bias.

### Conclusions

The small number of health professionals giving information and the extensive use of internet- and media-based sources highlight the need to address the quality of internet advice and guide women toward trustworthy sources of information during pregnancy. Even though internet and media sources seemed to have a positive impact on nutritional behavior, it was also associated with gaining below the GWG guidelines. Further research investigating how different information sources influence PA, GWG, and nutritional behaviors is needed.

## Abbreviations

BMI: body mass index
GWG: gestational weight gain
IOM: Institute of Medicine
OR: odds ratio
PA: physical activity
REK: Regional Committee for Medical and Health Research Ethics
